# Sex-stratified prognostic value of low skeletal muscle index in advanced non-small cell lung Cancer: a retrospective cohort study

**DOI:** 10.3389/fnut.2026.1816649

**Published:** 2026-07-20

**Authors:** Xin-qi Li, Yan-xu Wu, Yun-tao Lu, Ting Zhao, Ying-gang Zhu

**Affiliations:** Department of Pulmonary and Critical Care Medicine, Huadong Hospital, Fudan University, Shanghai, China

**Keywords:** advanced non-small cell lung cancer, bioelectrical impedance analysis, overall survival, sarcopenia, sex differences, skeletal muscle index

## Abstract

**Background:**

Low skeletal muscle mass is associated with poor outcomes in non-small cell lung cancer (NSCLC), but whether the prognostic association differs by sex remains unclear. We evaluated the sex-stratified association between low skeletal muscle index (SMI) and overall survival in patients with advanced NSCLC.

**Methods:**

We conducted a retrospective analysis of 270 patients with advanced (stage III-IV) non-small cell lung cancer selected from a prospective cohort (*n* = 403) at Huadong Hospital. Baseline skeletal muscle index (SMI) was measured by bioelectrical impedance analysis (BIA) and dichotomized using AWGS thresholds (men <7.0 kg/m^2^; women <5.7 kg/m^2^). Sex-stratified Cox proportional hazards regression was performed to assess the association between low SMI and overall survival, adjusting for age, disease stage, performance status, comorbidities, and treatment modality. Sensitivity analyses were performed.

**Results:**

During a median follow-up of 392 days, 145 deaths occurred (53.7% mortality). In males, low SMI was independently associated with increased mortality risk (adjusted HR = 1.67, 95% CI: 1.11–2.51, *p* = 0.013); estimates were essentially unchanged after additional adjustment for smoking and albumin (HR range 1.58–1.68; all *P* < = 0.029). In females, no statistically significant association was detected (adjusted HR = 0.81, 95% CI: 0.40–1.65, *p* = 0.564), noting the limited power of the female subgroup. The sex-by-SMI interaction was also not statistically significant (*P*-interaction = 0.115). Exploratory cutpoint analysis supports the applicability of the AWGS male threshold in males (7.0 kg/m2). Supplementary analyses using continuous SMI modeling, interaction testing, and sensitivity analyses excluding early deaths confirmed the robustness of these sex-stratified findings.

**Conclusion:**

BIA-defined low skeletal muscle mass (low SMI) was independently associated with poorer overall survival in male patients with advanced NSCLC, whereas no statistically significant association was detected in women. Given the limited sample size and statistical power of the female subgroup, these sex-stratified findings should be interpreted cautiously. As this study evaluated low muscle mass rather than full AWGS-defined sarcopenia, the findings should be interpreted accordingly. These results, derived from a single-center retrospective cohort, are hypothesis-generating and require external validation in larger, multi-center cohorts before sex-stratified interpretation can be applied in clinical practice.

## Introduction

Sarcopenia—the progressive loss of skeletal muscle mass and function—is highly prevalent in patients with non-small cell lung cancer (NSCLC), particularly in advanced stages, and has emerged as an important determinant of clinical outcomes. A comprehensive meta-analysis demonstrated that sarcopenia is associated with significantly worse overall survival (OS) in lung cancer across disease stages (1). In advanced NSCLC, imaging studies further indicate that distinct muscle phenotypes may carry differential prognostic implications: reduced skeletal muscle radiodensity (SMD), a marker of myosteatosis, has been independently associated with inferior survival, whereas associations with skeletal muscle index (SMI) have been variable after multivariable adjustment (2). These observations suggest that both measurement modality and case definition of “low muscle mass” critically influence observed prognostic effects.

Computed tomography (CT) at the third lumbar vertebral (L3) level is usually used as the reference standard for cross-sectional muscle quantification in oncology research. However, bioelectrical impedance analysis (BIA) is increasingly adopted in clinical practice because it is non-invasive, inexpensive, and suitable for repeated assessments. Studies in NSCLC populations report moderate-to-good correlation between BIA- and CT-derived muscle measures (3, 4). Importantly, systematic biases have been documented in specific clinical contexts. In newly diagnosed NSCLC patients, BIA tends to overestimate muscle mass and underestimate fat mass compared with CT, with wide limits of agreement that exceed clinically acceptable margins (3). Notably, the degree of overestimation was more pronounced among patients with poorer performance status, a characteristic common in advanced (stage III-IV) disease. The establishment of population-specific thresholds for bioelectrical impedance analysis-derived skeletal muscle index is particularly warranted in patients with advanced non-small cell lung cancer.

The 2019 consensus of the Asian Working Group for Sarcopenia (AWGS) established sex-specific SMI thresholds for BIA (men <7.0 kg/m^2^; women <5.7 kg/m^2^), which have been widely implemented in Asian clinical settings (5). Recently, the 2025 AWGS consensus update emphasized a paradigm shift from sarcopenia as a disease state toward the broader concept of muscle health across the life course, expanded diagnostic applicability to middle-aged adults, and simplified the diagnostic algorithm to focus on low muscle mass and low muscle strength (6). While these recommendations strengthen the conceptual and clinical relevance of SMI-based classification, the proposed thresholds were derived primarily from community-dwelling cohorts rather than oncology populations exposed to cancer-related metabolic and inflammatory stress. Outcome-driven, disease-specific cut-points have therefore been proposed. For example, a multi-center stage-III NSCLC study using CT identified sex-specific SMI thresholds independently associated with OS (7). However, the performance of previously published fixed CT-defined SMI thresholds has been inconsistent across clinical endpoints. In a cross-sectional study of lung cancer patients, several commonly used CT cut-points demonstrated limited discriminatory ability for predicting functional limitation, with area under the curve (AUC) values ranging from 0.52 to 0.59 (8). These findings highlight the context dependency of muscle mass thresholds and suggest that cut-points derived for one outcome or population may not generalize across clinical settings. Methodologically, maximally selected rank statistics have been applied to derive prognostic cut-points in oncology, including composite indices such as skeletal muscle gauge (SMG), demonstrating feasibility while also raising concerns regarding cohort-specific overfitting and limited external validity when independent validation or resampling procedures are not performed (9).

Biological sex introduces additional complexity. Baseline muscle mass, endocrine regulation, inflammatory responses, and body-composition trajectories differ between men and women, potentially modifying the prognostic impact of low muscle mass. Multiple oncology series suggest that adverse survival effects of sarcopenia are more pronounced in men (10–12). Recent prospective data using BIA in NSCLC demonstrated that obesity paradox–related survival advantages are sex-specific, with inflammatory mediation accounting for a greater proportion of the association between body composition and mortality in men than in women (13). Furthermore, high-resolution phenotyping in resectable NSCLC revealed distinct sex-specific depletion patterns: women with sarcopenia exhibited concurrent losses across muscle, fat, bone mineral content, and basal metabolic rate, whereas men more often showed relatively isolated muscle depletion (14). These observations suggest that the prognostic relevance and optimal thresholds of SMI may differ by sex, particularly in advanced disease characterized by systemic inflammation and catabolic burden. However, sex-stratified validation of AWGS-recommended BIA-derived SMI thresholds in advanced (stage III-IV) NSCLC remains limited.

Recognizing the unique muscle-wasting patterns in cancer, we explored the prognostic performance and empirical stability of AWGS-recommended SMI thresholds in advanced NSCLC. As an exploratory analysis, our objective was to evaluate whether alternative SMI cutpoints could improve the identification of patients with low skeletal muscle mass and enhance risk stratification in this vulnerable population.

## Methods

### Study design and patient selection

This retrospective cohort study (Ethics Approval No. 20210069) was conducted using data from a prospectively established cohort of 403 patients, with institutional waiver of informed consent (15).

Between January 2020 and June 2025, we enrolled 270 consecutive patients with histologically confirmed stage III-IV non-small cell lung cancer (NSCLC). Patients were excluded if they had small-cell lung cancer, stage I-II disease, missing SMI data, or did not receive any systemic therapy (chemotherapy, radiotherapy, targeted therapy, or immunotherapy). The final analytic cohort comprised 270 patients (191 males, 79 females).

### Body composition assessment

Whole-body composition was assessed at baseline using bioelectrical impedance analysis (InBody 570, Korea). Measurements were performed in the morning after an overnight fast (≥8 h) and before intravenous fluid administration. Participants wore light clothing and were instructed to empty their bladders before assessment.

BIA was selected over CT-derived skeletal muscle area for three reasons: (i) BIA permits standardized whole-body assessment of appendicular skeletal muscle mass on a single device, whereas not all advanced NSCLC patients had abdominal imaging at the L3 level within the pre-specified baseline window; (ii) BIA is non-invasive and radiation-free, allowing future serial monitoring during therapy in routine practice; and (iii) BIA-derived SMI is the modality on which the AWGS 2019 and 2025 consensus thresholds are explicitly defined, ensuring direct comparability with the AWGS reference framework.

Skeletal muscle index (SMI) was calculated as appendicular skeletal muscle mass divided by height squared (kg/m^2^). Low SMI was defined according to the Asian Working Group for Sarcopenia (AWGS) 2019 recommendations as <7.0 kg/m^2^ for men and <5.7 kg/m^2^ for women. These cutoffs were pre-specified based on established consensus guidelines for BIA-derived SMI. The 2025 update of the AWGS reaffirmed the BIA-based SMI cutoffs established in 2019, supporting their continued use in clinical research.

Importantly, this study assessed low muscle mass — one of the three AWGS 2019 sarcopenia diagnostic domains — rather than full clinical sarcopenia, which additionally requires impaired muscle strength (e.g., hand grip) and physical performance (e.g., gait speed). Strength and performance measures were not systematically collected in this cohort; we therefore used the terms ‘low SMI’ or ‘low muscle mass’ rather than ‘sarcopenia’ throughout. The implications of BIA-based muscle assessment, relative to computed tomography (the imaging-based reference standard), are addressed in the Discussion.

### Data collection

Baseline demographic, clinical, and laboratory data were extracted from electronic medical records. Variables included age, sex, height, body mass index, Eastern Cooperative Oncology Group (ECOG) performance status, disease stage, histological type, treatment modality, smoking status, comorbidities (hypertension, diabetes), Charlson comorbidity index, tumor markers (CEA, Cyfra21-1, SCC antigen, NSE), albumin, and molecular markers (EGFR, KRAS, ALK, ROS1).

Overall survival (OS) was defined as the time from diagnosis to death from any cause. Patients alive at last follow-up (June 2025) were censored. Vital status was ascertained through hospital records and telephone contact when necessary.

### Follow-ups

Patients were followed through outpatient visits and review of electronic medical records until June 2025. Vital status was ascertained through hospital records and, when necessary, telephone contact.

### Statistical analysis

Continuous variables were presented as mean ± standard deviation (SD) for normally distributed data or median [interquartile range (IQR)] for non-normally distributed data, assessed by the Shapiro–Wilk test. Categorical variables were presented as frequencies and percentages. Baseline characteristics were compared between males and females using independent t-tests or Wilcoxon rank-sum tests for continuous variables and chi-square tests or Fisher’s exact tests for categorical variables.

Survival curves were estimated using the Kaplan–Meier method and compared using log-rank tests. Univariable Cox proportional hazards regression was performed to assess the association between low SMI and OS, stratified by sex.

Multivariable Cox proportional hazards regression models were fitted separately for males and females to estimate the independent association between low SMI and OS. Variable selection was based on established prognostic factors and confounding potential, not statistical significance. A minimally adjusted model (Model A) including only age and stage was also fitted for both sexes to assess robustness across adjustment levels. For males (191 patients, 106 events), the fully adjusted model (Model B) included age (continuous), stage (III vs. IV), ECOG performance status (0–1 vs. > =2), Charlson comorbidity index (continuous), and treatment type (targeted therapy, immunotherapy/combination, chemotherapy/radiotherapy), in addition to SMI category (events per variable [EPV] = 15.14). For females (79 patients, 39 events), the fully adjusted model included age (continuous), stage (III vs. IV), and ECOG performance status (0–1 vs. > =2), in addition to SMI category (EPV = 9.75). Different adjustment sets were pre-specified based on EPV considerations to maintain adequate EPV (>9) and avoid overfitting. The proportional hazards assumption was assessed using Schoenfeld residuals. Model discrimination was assessed using Harrell’s C-statistic.

### Exploratory cutpoint analysis

As an exploratory analysis, we applied maximally selected rank statistics with bootstrap resampling (1,000 iterations) to derive data-driven optimal SMI cutpoints in the 270-patient cohort. The surv_cutpoint function from the survminer R package was used to identify cutpoints that maximally separated survival curves. Bootstrap resampling assessed the stability of identified cutpoints by repeating the derivation procedure on 1,000 bootstrap samples.

### Supplementary analyses

Five supplementary analyses were performed. First, SMI was modeled as a continuous variable (per 1 standard deviation decrease) using sex-specific SDs (males: 0.74 kg/m2; females: 0.72 kg/m2). Second, interaction between sex and SMI (both binary and continuous) was tested in the combined cohort using likelihood ratio tests. Third, sensitivity analyses excluded patients who died within 30 days of diagnosis to address potential reverse causation. Fourth, additional sensitivity analyses were performed to evaluate potential residual confounding by smoking exposure and nutritional/inflammatory burden. Beyond the primary multivariable model (Model B), Model C additionally adjusted for smoking status (current or former vs. never), Model D additionally adjusted for serum albumin (continuous, g/L), and Model E additionally adjusted for both smoking and albumin. In the female subgroup, only Model D was estimable because smoking adjustment was not feasible due to the very low prevalence of ever/current smokers (5/79, 6.3%), which produced sparse-data instability with inflated standard errors. Models D and E included 189 male / 78 female patients due to listwise deletion of 3 missing serum albumin values (1.1%). Fifth, *post-hoc* power analyses were performed in the female subgroup (*n* = 79, 39 events) to assess statistical power for detecting effect sizes comparable to those observed in males. Power calculations were based on the Schoenfeld closed-form approximation for two-group Cox proportional hazards models, using the formula: $\text{power}=\Phi (\|\log(\mathrm{HR})\|\sqrt{d\cdot p\cdot (1-p})-z_{1-\alpha /2})$ . Where d is the number of events, p is the prevalence of low SMI, and α = 0.05 (two-sided). This analysis allowed estimation of both the statistical power to detect the male-observed hazard ratio and the minimum detectable hazard ratios at conventional 80 and 90% power thresholds.

**Figure 1:**
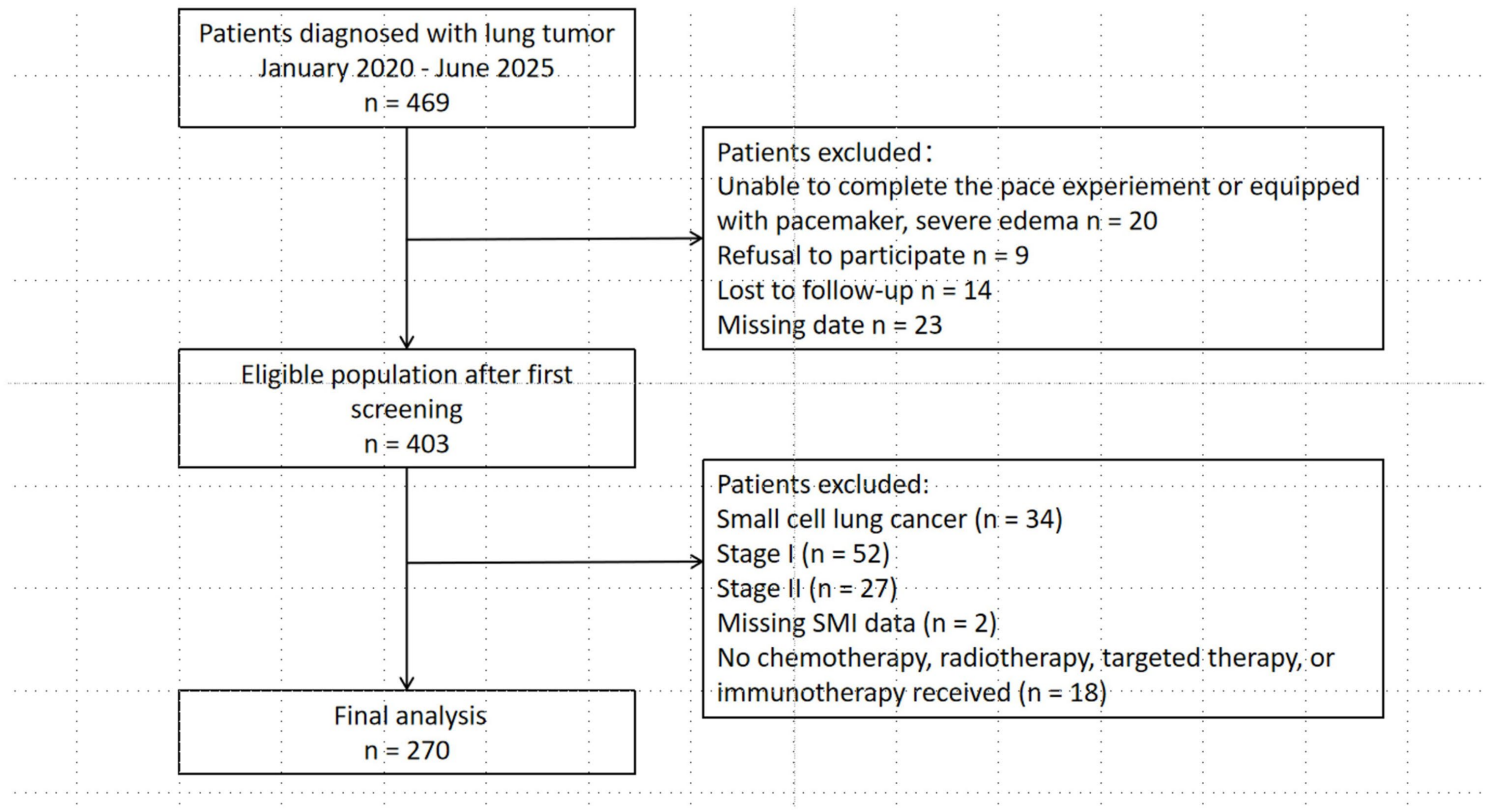
Enrollment flow diagram. Flow diagram illustrating the patient selection process. From 469 patients initially screened, 66 were excluded in the first round (technical/follow-up reasons), leaving 403 eligible patients. An additional 133 were excluded in the second round (clinical criteria), resulting in a final analytic cohort of 270 patients (191 males, 79 females) with stage III-IV non-small cell lung cancer who received systemic therapy.

All statistical tests were two-sided, and *p* < 0.05 was considered statistically significant. Analyses were performed using R version 4.5.0 (R Foundation for Statistical Computing, Vienna, Austria) with the survival package (Figure 1).

## Results

### Study population and baseline characteristics

The study included 270 patients with advanced NSCLC (191 males [70.7%], 79 females [29.3%]). Mean age was 71.8 +/− 8.4 years. Low SMI prevalence was 43.5% in males (83/191) and 32.9% in females (26/79) (*p* = 0.141). Males and females differed significantly in height, SMI, histological type (males: more squamous cell carcinoma; females: more adenocarcinoma), treatment modality (females: more targeted therapy), and smoking status (males: 55.5% current/former smokers; females: 6.3%), and EGFR mutation status (males: 25.1% mutant; females: 64.6%) (all *p* < 0.001). Detailed baseline characteristics are presented in Table 1.

**Table 1:** Baseline characteristics of patients by sex.

Characteristic	Overall (*N* = 270)	Male (*n* = 191)	Female (*n* = 79)	*p*-value
Demographics				
Age, years	71.78 ± 8.41	71.35 ± 7.40	72.82 ± 10.46	0.190
Height, cm	165.84 ± 7.70	169.23 ± 5.87	157.67 ± 4.97	**<0.001**
Body mass index, kg/m^2^	22.01 ± 3.00	21.78 ± 2.94	22.55 ± 3.09	0.056
Low (<18.5)	34 (12.6)	25 (13.1)	9 (11.4)	0.070
Normal (18.5–24.9)	163 (60.4)	122 (63.9)	41 (51.9)	
High (≥25.0)	73 (27.0)	44 (23.0)	29 (36.7)	
Body composition				
Skeletal muscle index, kg/m^2^	6.73 ± 0.88	7.04 ± 0.74	5.97 ± 0.72	**<0.001**
AWGS-defined sarcopenia				0.141
Low SMI	109 (40.4)	83 (43.5)	26 (32.9)	
Normal/High SMI	161 (59.6)	108 (56.5)	53 (67.1)	
Performance status				
ECOG performance status				0.337
0	49 (18.1)	36 (18.8)	13 (16.5)	
1	144 (53.3)	103 (53.9)	41 (51.9)	
2	43 (15.9)	26 (13.6)	17 (21.5)	
3	27 (10.0)	22 (11.5)	5 (6.3)	
4	7 (2.6)	4 (2.1)	3 (3.8)	
Disease characteristics				
Clinical stage				0.401
III	62 (23.0)	47 (24.6)	15 (19.0)	
IV	208 (77.0)	144 (75.4)	64 (81.0)	
Histological type				**<0.001**
Adenocarcinoma	191 (70.7)	120 (62.8)	71 (89.9)	
Squamous cell carcinoma	76 (28.1)	69 (36.1)	7 (8.9)	
Large cell carcinoma	3 (1.1)	2 (1.0)	1 (1.3)	
Treatment Modality				**<0.001**
Targeted therapy	152 (56.3)	84 (44.0)	68 (86.1)	
Immunotherapy/combination	92 (34.1)	84 (44.0)	8 (10.1)	
Chemotherapy/radiotherapy	26 (9.6)	23 (12.0)	3 (3.8)	
Risk factors and comorbidities				
Smoking status				**<0.001**
Current/former smoker	111 (41.1)	106 (55.5)	5 (6.3)	
Never smoker	159 (58.9)	85 (44.5)	74 (93.7)	
Hypertension	134 (49.6)	93 (48.7)	41 (51.9)	0.729
Type 2 diabetes mellitus	59 (21.9)	47 (24.6)	12 (15.2)	0.123
Charlson comorbidity index	8.41 ± 2.27	8.37 ± 2.35	8.52 ± 2.09	0.617
Laboratory parameters				
Tumor markersᵃ, median [IQR]				
CEA, ng/mL	8.20 [3.45–32.40]	6.30 [3.27–22.53]	13.70 [4.55–47.95]	**0.022**
Cyfra21-1, ng/mL	4.50 [2.94–10.72]	4.60 [3.12–12.04]	3.94 [2.64–9.18]	0.167
SCC antigen, ng/mL	1.25 [0.80–1.90]	1.30 [0.90–2.25]	0.90 [0.70–1.40]	**0.001**
NSE, ng/mL	15.40 [12.50–20.10]	15.10 [12.20–19.85]	15.95 [13.88–20.43]	0.161
Albumin, g/L	38.99 ± 5.36	38.78 ± 5.28	39.51 ± 5.55	0.313
Molecular markers				
EGFR				**<0.001**
Wild-type	171 (63.3)	143 (74.9)	28 (35.4)	
Mutant	99 (36.7)	48 (25.1)	51 (64.6)	
KRASᵇ				0.073
Wild-type	256 (94.8)	178 (93.2)	78 (98.7)	
Mutant	14 (5.2)	13 (6.8)	1 (1.3)	
ALKᵇ				0.293
Wild-type	269 (99.6)	191 (100.0)	78 (98.7)	
Mutant	1 (0.4)	0 (0.0)	1 (1.3)	
ROS1ᵇ				1.000
Wild-type	268 (99.3)	189 (99.0)	79 (100.0)	
Mutant	2 (0.7)	2 (1.0)	0 (0.0)	

AWGS, Asian Working Group for Sarcopenia; BMI, body mass index; CEA, carcinoembryonic antigen; ECOG, Eastern Cooperative Oncology Group; IQR, interquartile range; NSE, neuron-specific enolase; SCC, squamous cell carcinoma antigen; SD, standard deviation; SMI, skeletal muscle index. Sarcopenia was defined according to AWGS criteria using sex-specific SMI cutoffs: <7.0 kg/m^2^ for males and <5.7 kg/m^2^ for females. Data are presented as mean (SD) for normally distributed continuous variables, median [interquartile range] for non-normally distributed continuous variables, or *n* (%) for categorical variables. *p*-values were calculated using an independent *t*-test or Wilcoxon rank-sum test for continuous variables and chi-square test or Fisher’s exact test for categorical variables. ᵃTumor markers (CEA, Cyfra21-1, SCC, NSE) showed non-normal distributions (Shapiro–Wilk test, all *P* < 0.001) and were compared using the Wilcoxon rank-sum test. 𝑿or mutations with low event counts (ALK, ROS1, KRAS), Fisher’s exact test was used due to expected cell frequencies <5. Bold values indicate statistical significance (*p* < 0.05).

### Follow-up and survival

During a median follow-up of 392 days (range: 1–1,826 days), 145 deaths occurred, yielding an overall mortality rate of 53.7%. Mortality rates were 55.5% in males (106/191) and 49.4% in females (39/79).

### Univariable analysis

**Figure 2:**
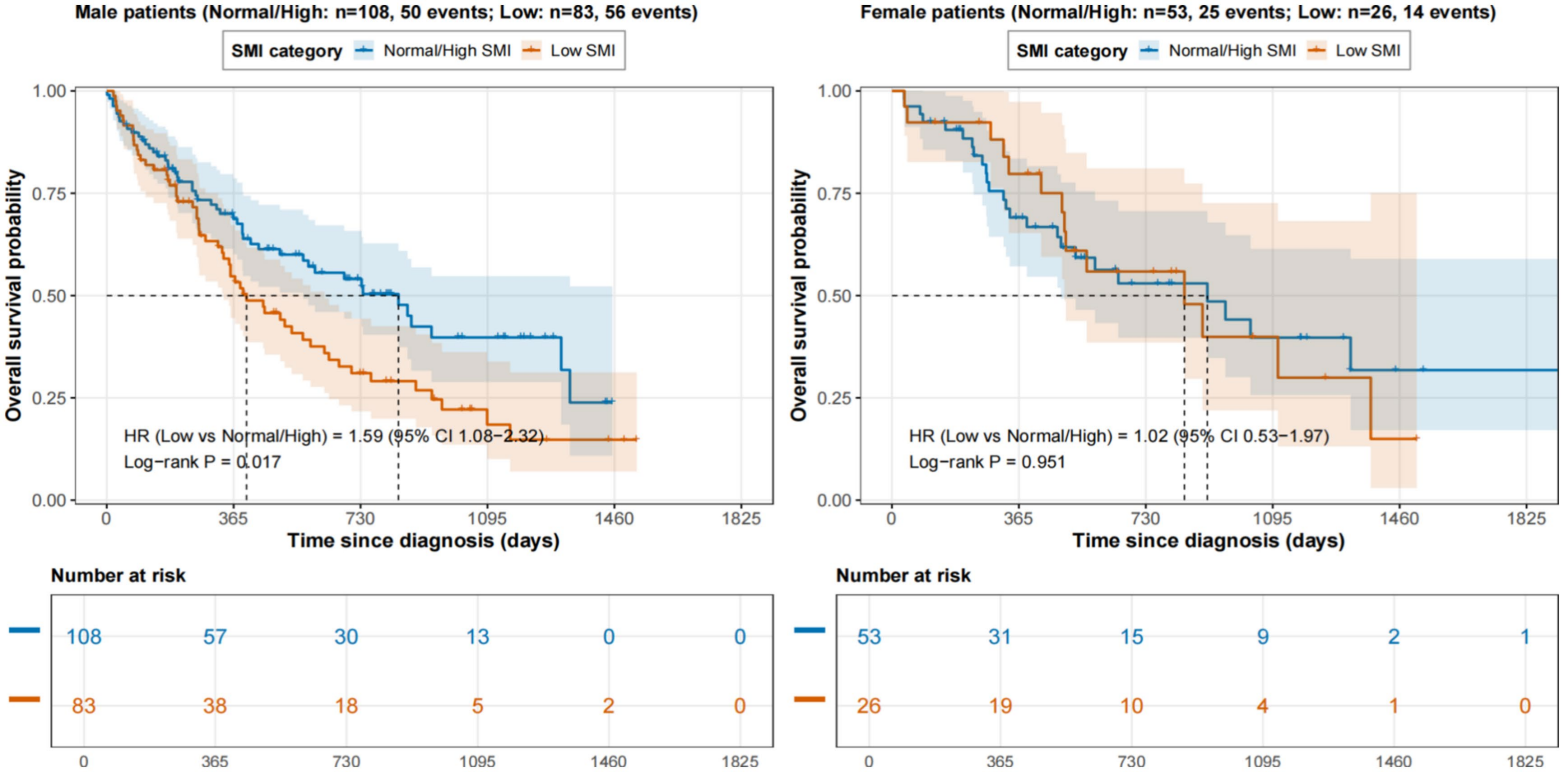
Kaplan–Meier survival curves stratified by sex and SMI category. Kaplan–Meier curves showing overall survival probability by AWGS-defined SMI category (low vs. normal/high) stratified by sex. **(A)** Male patients (*n* = 191): Low SMI was associated with significantly shorter survival compared to normal/high SMI (median 402 vs. 839 days, log-rank *p* = 0.017). **(B)** Female patients (*n* = 79): No statistically significant difference in survival was observed between SMI groups (median 841 vs. 907 days, log-rank *p* = 0.951). Shaded areas represent 95% confidence intervals. Numbers at risk are shown below each plot. Sarcopenia was defined using sex-specific AWGS cutoffs: <7.0 kg/m^2^ for males and <5.7 kg/m^2^ for females.

In univariable Cox regression, low SMI was significantly associated with increased mortality risk in males (HR = 1.59, 95% CI: 1.08–2.32, *p* = 0.018; Table 2) but not in females (HR = 1.02, 95% CI: 0.53–1.97, *p* = 0.950; Table 2). Kaplan–Meier analysis showed significantly worse survival in males with low SMI (median survival: 402 days for low SMI vs. 839 days for normal/high SMI, log-rank *p* = 0.017), while no statistically significant survival difference was observed in females (median survival: 841 days for low SMI vs. 907 days for normal/high SMI, log-rank *p* = 0.951; Table 3 and Figure 2).

**Table 2:** Sex-stratified univariable Cox regression analysis for overall survival.

SMI category	Male (*n* = 191)				Female (*n* = 79)			
	*N*	Events	HR (95% CI)	*P*	*N*	Events	HR (95% CI)	*P*
Normal/High SMI	108	50	1.00 (Ref)	-	53	25	1.00 (Ref)	–
Low SMI	83	56	1.59 (1.08–2.32)	0.018	26	14	1.02 (0.53–1.97)	0.950

HR, hazard ratio; CI, confidence interval; SMI, skeletal muscle index; AWGS, Asian Working Group for Sarcopenia. Low SMI is defined as <7.0 kg/m^2^ for males and <5.7 kg/m^2^ for females. *P-*values from the Wald test. Proportional hazards assumption assessed using Schoenfeld residuals (global test *P* > 0.05 for both sexes).

**Table 3:** Median overall survival by sex and SMI category (Kaplan–Meier analysis).

SMI category	Male		Female	
	*N*	Median (95% CI), days	*N*	Median (95% CI), days
Normal/High SMI	108	839 (565-NR)	53	907 (486-NR)
Low SMI	83	402 (336–626)	26	841 (496-NR)
Log-rank test		***P* = 0.017**		***P* = 0.951**

SMI, skeletal muscle index; CI, confidence interval. Low SMI is defined as <7.0 kg/m^2^for males and <5.7 kg/m^2^ for females according to AWGS criteria. *P-*values from the log-rank test comparing survival curves between low and normal/high SMI groups within each sex.

### Multivariable analysis

Sex-stratified multivariable Cox regression confirmed the independent prognostic value of low SMI in males but not in females (Table 4).

**Table 4:** Multivariable Cox regression analysis for overall survival.

Variable	Male		Female	
	Model A	Model B	Model A	Model B
	HR (95% CI)	HR (95% CI)	HR (95% CI)	HR (95% CI)
	*P-*value	*P-*value	*P-*value	*P-*value
SMI (AWGS criteria)				
Normal/High	1.00 (Ref)	1.00 (Ref)	1.00 (Ref)	1.00 (Ref)
Low	**1.63 (1.10–2.42)**	**1.67 (1.11–2.51)**	0.89 (0.46–1.72)	0.81 (0.40–1.65)
	***p* = 0.016**	***p* = 0.013**	*p* = 0.719	*p* = 0.564
Age (per year)	1.00 (0.98–1.03)	1.00 (0.97–1.03)	**1.05 (1.01–1.09)**	**1.05 (1.01–1.09)**
	*p* = 0.928	*p* = 0.890	***p* = 0.007**	*p* = 0.008
Stage				
III	1.00 (Ref)	1.00 (Ref)	1.00 (Ref)	1.00 (Ref)
IV	**3.16 (1.76–5.68)**	**2.25 (1.11–4.58)**	2.26 (0.86–5.92)	2.15 (0.81–5.70)
	***p* < 0.001**	***p* = 0.025**	*p* = 0.099	*p* = 0.125
ECOG performance status				
0–1	—	1.00 (Ref)	—	1.00 (Ref)
≥2	—	1.43 (0.94–2.19)	—	1.32 (0.64–2.72)
		***p* = 0.098**		*p* = 0.460
Charlson comorbidity index (per point)	—	1.09 (0.96–1.23)	—	—
		*p* = 0.174		
Treatment type				
A (Targeted)	—	1.00 (Ref)	—	—
B (Immuno/Combined)	—	1.20 (0.77–1.88)	—	—
		*p* = 0.426		
C (Chemo/Radio)	—	1.05 (0.54–2.07)	—	—
		*p* = 0.881		
Model summary				
Sample Size	*N* = 191	*N* = 191	*N* = 79	*N* = 79
Events	106	106	39	39
EPV	35.33	15.14	13.00	9.75
C-statistic	0.608	0.643	0.656	0.655
PH assumption (P)	0.573	0.239	0.435	0.353

HR, hazard ratio; CI, confidence interval; SMI, skeletal muscle index; AWGS, Asian Working Group for Sarcopenia; ECOG, Eastern Cooperative Oncology Group; EPV, events per variable. Model A adjusted for age and stage. Model B: for males (106 events), adjusted for age, stage, ECOG performance status, Charlson comorbidity index, and treatment type (EPV = 15.14); for females (39 events), adjusted for age, stage, and ECOG performance status (EPV = 9.75). Different adjustment sets were pre-specified based on EPV considerations. Low SMI is defined as <7.0 kg/m^2^ for males and <5.7 kg/m^2^ for females. Reference categories: SMI (Normal/High), Stage (III), ECOG (0–1), Treatment (Targeted therapy). *P-*values from the Wald test. Proportional hazards assumption satisfied for all models (global test *P* > 0.05). Bold values indicate statistical significance (*p* < 0.05).

In males (*N* = 191, 106 events), low SMI was significantly associated with increased mortality risk in both the minimally adjusted model (Model A: HR = 1.63, 95% CI: 1.10–2.42, *p* = 0.016) and the fully adjusted model (Model B: HR = 1.67, 95% CI: 1.11–2.51, *p* = 0.013). The fully adjusted model indicated that low SMI was independently associated with a 67% higher hazard of mortality after adjusting for age, stage, performance status, comorbidities, and treatment modality. Among other covariates, only stage IV disease was significantly associated with mortality (HR = 2.25, 95% CI: 1.11–4.58, *p* = 0.025).

In females (*N* = 79, 39 events), no statistically significant association between low SMI and mortality was detected in either the minimally adjusted model (Model A: HR = 0.89, 95% CI: 0.46–1.72, *p* = 0.719) or the fully adjusted model (Model B: HR = 0.81, 95% CI: 0.40–1.65, *p* = 0.564). Age was the only significant prognostic factor in the female model (HR = 1.05, 95% CI: 1.01–1.09, *p* = 0.008).

The consistency of SMI effects across adjustment levels (males: HR = 1.63 vs. 1.67; females: HR = 0.89 vs. 0.81) demonstrated robustness. Model discrimination was moderate for both sexes (male Model B: C-statistic = 0.643; female Model B: C-statistic = 0.655). The proportional hazards assumption was satisfied for all models (global test *p* > 0.05).

### Exploratory cut-off point analysis

Exploratory cutpoint analysis identified an empirical optimal SMI cutpoint of 6.9 kg/m^2^ in males (bootstrap median 7.0 kg/m^2^; empirical 95% interval 6.2–7.8 kg/m^2^, Supplementary Table S1 and Supplementary Figures S1, S2), closely aligning with the AWGS guideline (7.0 kg/m^2^). Bootstrap resampling (1,000 iterations) produced valid estimates in all iterations, indicating high stability of the male threshold.

In females, the empirical optimal cutpoint was 5.1 kg/m^2^ (bootstrap median 5.4 kg/m^2^; empirical 95% interval 4.9–6.8 kg/m^2^, Supplementary Table S2 and Supplementary Figures S3, S4). The AWGS value (5.7 kg/m^2^) lies within the female bootstrap interval, though the broader distribution indicates greater uncertainty in the female subgroup, likely reflecting the smaller sample and event counts. For consistency with established guidelines and to facilitate clinical implementation, we retained the AWGS cutoffs (7.0 kg/m^2^ for males, 5.7 kg/m^2^ for females) for all primary and supplementary analyses.

### Supplementary analyses

When SMI was modeled as a continuous variable, associations showed directional consistency with binary analysis (Supplementary Table S3 and Supplementary Figure S5). In males, each 1 SD decrease in SMI was associated with a 14% increased mortality risk (HR = 1.14, 95% CI: 0.92–1.42, *p* = 0.230). In females, no statistically significant association was observed (HR = 0.90, 95% CI: 0.62–1.30, *p* = 0.573, Supplementary Table S3 and Supplementary Figure S5).

Formal interaction testing showed a trend toward sex differences in the SMI-survival association. For binary SMI, the interaction term had *p* = 0.120 (Wald test) and *p* = 0.115 (likelihood ratio test). For continuous SMI, interaction *p*-values were 0.551 and 0.554, respectively.

Sensitivity analysis excluding 10 patients who died within 30 days (all male) strengthened the male findings (HR = 1.82, 95% CI: 1.19–2.78, *p* = 0.006), demonstrating that results were not driven by reverse causation. Female results were unchanged as no early deaths occurred in this subgroup.

Additional sensitivity analyses evaluating residual confounding by smoking exposure and nutritional/inflammatory burden demonstrated stable results in males across all models (HR range 1.58–1.68, all *p* ≤ 0.05), supporting robustness of the primary findings. Adjustment for smoking status and/or serum albumin did not materially alter the observed association between low SMI and mortality in males. In females, the null association persisted after albumin adjustment (HR = 0.88, 95% CI: 0.43–1.79, *p* = 0.726). Smoking-adjusted models were not estimable in females because only 5 of 79 women (6.3%) were ever/current smokers, resulting in sparse-data instability with inflated standard errors (Supplementary Table S6).

To evaluate whether the absence of statistical association in females reflected limited statistical power, post-hoc power analyses were performed using the Schoenfeld approximation for two-group Cox proportional hazards models. In the female subgroup (*n* = 79, 39 events, low SMI prevalence = 32.9%), statistical power to detect the male-observed HR of 1.67 was only 32.5%. The minimum detectable HR at 80% power was approximately 2.60. These findings suggest that the female subgroup analysis should be interpreted as “no statistically significant association detected” rather than evidence of absence of a true association (Supplementary Table S7).

## Discussion

### Sex-stratified prognostic association of low SMI

This retrospective cohort study of 270 patients with advanced NSCLC demonstrates that low skeletal muscle index (SMI) measured by bioelectrical impedance analysis was an independent prognostic factor for overall survival in males. In females, no statistically significant association was detected. In sex-stratified multivariable Cox regression, males with low SMI (<7.0 kg/m^2^) had a 67% increased risk of mortality (HR = 1.67, 95% CI: 1.11–2.51, *p* = 0.013) after adjusting for age, disease stage, performance status, comorbidities, and treatment modality. In contrast, no statistically significant association was observed in females (HR = 0.81, 95% CI: 0.40–1.65, *p* = 0.564). Although the formal sex-by-SMI interaction test did not reach statistical significance (binary SMI: *p* = 0.120; continuous SMI: *p* = 0.551), likely reflecting limited power for interaction testing in our sample, the directionally divergent hazard ratios between sexes (HR 1.67 vs. 0.81) support sex-stratified reporting as suggestive evidence of potential effect heterogeneity, rather than definitive effect modification. These sex-stratified findings were robust across multiple sensitivity analyses, including continuous SMI modeling, formal interaction testing, and exclusion of early deaths.

### Applicability of AWGS cutoffs in cancer populations

A notable strength of this study is the concordance between the empirically derived male SMI cutpoint (bootstrap median 7.0 kg/m^2^) and the Asian Working Group for Sarcopenia (AWGS) 2019 recommendation (7.0 kg/m^2^). This alignment is noteworthy given that AWGS cutoffs were originally derived from community-dwelling older adults rather than cancer populations. The bootstrap analysis (1,000 iterations) demonstrated stability of the male threshold (95% CI: 6.2–7.8 kg/m^2^) with a 100% success rate, supporting the applicability of the AWGS criteria in advanced NSCLC.

In females, the empirically derived cutpoint (bootstrap median 5.4 kg/m^2^) was lower than the AWGS guideline (5.7 kg/m^2^), although the AWGS value fell within the bootstrap confidence interval (4.9–6.8 kg/m^2^), suggesting reasonable concordance. The broader distribution in females likely reflects the smaller sample size (*n* = 79) and fewer events (*n* = 39), resulting in greater statistical uncertainty. The AWGS cutoff may therefore be retained for consistency with existing guidelines, pending validation in larger cohorts.

### Rationale for disease-specific and data-driven cutpoints

The rationale for exploring data-driven, disease-specific cut-points warrants emphasis. The AWGS 2019 consensus established BIA-derived SMI cut-points (<7.0 kg/m^2^ for men and <5.7 kg/m^2^ for women), derived primarily from Asian community-dwelling reference populations (5); these thresholds have been retained in subsequent consensus updates (6). While AWGS guidelines have been validated in prospective community-based cohorts of adults aged ≥75 years (16), they were designed for general population sarcopenia diagnosis rather than cancer-specific risk stratification in patients exposed to intense catabolic and inflammatory stress. Recent work in advanced NSCLC has identified disease-specific CT-based SMI thresholds (men <45.1 cm^2^/m^2^; women <38.7 cm^2^/m^2^) associated with overall survival (HR 1.40, *p* = 0.038) (7), suggesting that population-specific specification may enhance prognostic discrimination. Methodologically, maximally selected rank statistics have been applied in advanced NSCLC to define imaging-based thresholds, including for skeletal muscle gauge (9) and myosteatosis markers (17). These data-driven approaches help address a recognized limitation: several commonly cited CT-based cutpoints, when tested prospectively for their ability to predict functional limitation in lung cancer patients, demonstrated limited discriminatory ability (area under the curve 0.516–0.592) (8), raising questions about whether fixed thresholds derived in one context generalize to other clinical endpoints or populations.

### Methodological considerations of AWGS-derived thresholds

Important methodological considerations regarding AWGS-derived thresholds merit clarity. The AWGS 2019 consensus established sex-specific SMI cut-points based on regional population data and expert consensus derived primarily from Asian community-dwelling cohorts (5). Validation studies in diverse clinical populations have revealed device-specific variation: A Japanese population demonstrated that AWGS BIA cutoffs (<7.0 kg/m^2^ in men; <5.7 kg/m^2^ in women) were consistent with DXA and TANITA multi-frequency BIA measurements, whereas raw InBody values required device-specific recalibration (18). In prospective community-dwelling cohorts, the AWGS 2019 update was associated with superior discrimination of mortality risk compared with the earlier 2014 criteria in elderly adults (16). However, performance across clinical contexts varies: comparative analyses in heart failure populations indicated that AWGS 2019 criteria identified more patients overall (24.0% vs. 21.1% with 2014 criteria) yet demonstrated similar prognostic performance (19). These observations suggest that while AWGS thresholds provide a validated, consensus-based reference point with demonstrated prognostic utility in general populations, their derivation from community rather than oncology cohorts indicates they may not fully capture the heterogeneity of cancer-related muscle depletion patterns. Additionally, alternative biomarkers—serologic indices such as the creatinine/cystatin C ratio and sarcopenia index—have been validated against AWGS definitions in advanced NSCLC (*n* = 579), demonstrating comparable prognostic performance, with hazard ratios ranging from 1.55 to 1.76 across definitions (20). These findings further support the relevance of AWGS-based classification in lung cancer populations and indicate that AWGS criteria serve as a meaningful reference framework for cancer-specific prognostic evaluation. The convergence between our empirically derived male cutpoint and the AWGS threshold, supported by a narrow bootstrap distribution, provides evidence consistent with the applicability of the AWGS value in advanced NSCLC; however, this finding is specific to our single-center cohort and requires independent external replication in other advanced NSCLC populations.

### Context within NSCLC skeletal muscle literature

These findings address an important context within the broader NSCLC sarcopenia literature. A meta-analysis of 13 cohort studies comprising 1,810 participants reported that sarcopenia was associated with worse overall survival in NSCLC overall (HR 2.57, 95% CI 1.79–3.68) (1). Stage-specific associations varied, with hazard ratios of 3.23 (95% CI 1.68–6.23) for early-stage disease and 2.19 (95% CI 1.14–4.24) for stage III-IV disease (1). Collectively, these meta-analytic summaries and individual cohorts point to a consistent direction: sarcopenia is associated with adverse outcomes in NSCLC across disease stages. Consistent with this trend, meta-analytic evidence from resected NSCLC populations found sarcopenia to be an independent adverse prognostic factor (HR 2.85, 95% CI 1.67–4.86) (21). A large retrospective study of resected NSCLC (n = 328) similarly identified sarcopenia as an independent predictor (*p* = 0.019), with median age 71 years and 55.8% prevalence of sarcopenia (22). However, important heterogeneity exists in how this prognostic signal manifests. When measurement modality and specific phenotype are examined, a more nuanced picture emerges: in advanced NSCLC specifically, CT-derived skeletal muscle radiodensity (SMD), a marker of myosteatosis, was significantly associated with mortality risk (2), whereas skeletal muscle index alone did not achieve statistical significance in multivariable adjustment in the same cohort (2). Recent studies in advanced NSCLC further demonstrate that intramuscular adipose tissue and SMD together identify patients at elevated mortality risk (17). These observations underscore that abnormalities in skeletal muscle composition are important determinants of NSCLC prognosis; however, the observed associations may vary according to the specific muscle-related phenotype assessed (e.g., low muscle mass versus muscle quality), the measurement modality, and disease stage.

### Sensitivity analyses and robustness

Our study contributes to this literature by employing rigorous sex-stratified multivariable analysis with pre-specified adjustment sets based on events per variable (EPV) considerations, formal interaction testing, and multiple sensitivity analyses. The consistency of our findings across binary and continuous SMI modeling, and the strengthening of male associations after excluding early deaths (HR = 1.82, 95% CI: 1.19–2.78, *p* = 0.006), support the robustness and validity of the observed sex differences.

Additional sensitivity analyses demonstrated stable male associations after further adjustment for smoking status and serum albumin, suggesting that the observed prognostic effect of low SMI in males was not materially explained by residual confounding from smoking exposure or nutritional/inflammatory burden. In females, the null association persisted after albumin adjustment, although smoking-adjusted models were not estimable because smoking exposure was rare in women, resulting in sparse-data instability. Furthermore, the female subgroup included only 39 events and had limited statistical power, with only 32.5% power to detect the male-observed hazard ratio. Therefore, the absence of statistical significance in females should be interpreted cautiously, and a modest association cannot be excluded.

### Sex differences and biological plausibility

The observed sex-stratified findings warrant careful interpretation within the context of existing literature and potential biological explanations. Several studies in solid tumors have reported sex-stratified associations between muscle-related phenotypes and survival, with stronger associations observed in men than in women. Early-stage NSCLC studies demonstrated SMI-associated survival differences in men but not women when measurements were obtained at specific anatomic levels (thoracic vertebrae T5) (10). In a cohort of 215 male patients with pathological stage I NSCLC, sarcopenia was associated with significantly shorter median overall survival (32 months) (12). However, female patients were not included in the analysis. Similarly, in a retrospective cohort of 1,054 patients undergoing gastrectomy for gastric cancer, low skeletal muscle index independently predicted 5-year overall survival in men (HR 2.51, 95% CI 1.73–3.63) but not in women (11). Similar sex-differential associations emerged in colorectal cancer, where low abdominal skeletal muscle mass correlated with mortality in men but showed no statistically significant association in women (23). Cholangiocarcinoma studies similarly found that CT-measured skeletal muscle indices predicted survival in males only (24).

If true sex-related heterogeneity exists, several biological mechanisms may potentially contribute to these observed sex-stratified findings (25, 26). Although these mechanisms remain speculative in the context of the present study, prior research has proposed several potential explanations. First, sex hormones may modulate the relationship between muscle mass and cancer prognosis. Testosterone plays a critical role in maintaining muscle mass and anabolic metabolism in males (25, 27). Cancer-related cachexia and systemic inflammation may accelerate testosterone decline, leading to more rapid muscle loss (28, 29) and metabolic derangement in males compared with females (26). Conversely, mechanistic evidence indicates that estrogen modulates inflammatory and apoptotic signaling in skeletal muscle, which may partially mitigate muscle loss and contribute to sex-specific differences in the prognostic significance of low muscle mass (30–33). Second, recent body composition phenotyping and analysis of circulating inflammatory mediators in advanced NSCLC suggest that systemic inflammation may mediate the relationship between body composition and mortality differentially by sex: inflammatory pathways accounted for approximately 31% of the body composition–mortality association in men versus 20% in women (13). This sex-differential inflammatory mediation may be partly explained by distinct patterns of body composition depletion: a recent propensity-matched analysis of early-stage NSCLC patients (*n* = 460) found that men with sarcopenia displayed primarily isolated skeletal muscle depletion with relative preservation of adipose tissue and bone mineral content, whereas women demonstrated concurrent losses across muscle, fat, and mineral compartments (14). Although this body composition phenotyping was observed in early-stage NSCLC patients, the heightened systemic inflammation and catabolic burden characteristic of advanced lung cancer may exacerbate the underlying compartmental differences between sexes, potentially contributing to the sex-stratified findings observed in the present study. Besides, males typically have higher baseline muscle mass and lower body fat percentage compared with females (34). A given absolute reduction in muscle mass may therefore represent a more severe physiological perturbation in males, potentially contributing to more pronounced functional impairment and adverse outcomes. Third, sex differences in tumor biology and treatment—females in this cohort had more adenocarcinoma and greater use of targeted therapy—could attenuate or mask any adverse prognostic effect of low SMI. Moreover, the female subgroup was small (*n* = 79, 39 events), yielding a wide CI (HR 0.81, 95% CI 0.40–1.65) and limited power to detect modest effects. *Post-hoc* power analysis demonstrated only 32.5% power to detect the effect size observed in males. Therefore, the lack of a statistically significant association in females should be interpreted cautiously and requires confirmation in larger cohorts.

### Clinical implications

From a practical clinical standpoint, these observations suggest that AWGS-recommended SMI cutpoints warrant further evaluation as a risk-stratification tool for male patients with advanced NSCLC in settings where standardized BIA capacity exists. However, given the single-center retrospective design and absence of external validation, these findings should be viewed as hypothesis-generating, and routine clinical implementation would be premature. BIA offers potential advantages over CT-based approaches for routine clinical monitoring, including non-invasive assessment, lower cost, and feasibility for serial measurement during treatment. However, clinical application requires rigorous attention to measurement standardization and device-specific characteristics. BIA protocols should include overnight fasting, consistent timing, and device-specific calibration (18). Furthermore, clinicians should be aware that BIA-derived measurements may overestimate muscle mass relative to CT reference standards, particularly in patients with poor performance status—a characteristic common in advanced disease (3). Importantly, the documented direction of BIA-CT measurement bias in advanced NSCLC is overestimation of muscle mass, particularly in patients with poor performance status. This non-differential measurement error would, if anything, bias our hazard-ratio estimates toward the null, suggesting that the true prognostic effect of low muscle mass in male patients with advanced NSCLC may be underestimated rather than overestimated by our analysis. The robustness of the male SMI-survival association across all sensitivity models is consistent with this interpretation. These measurement considerations suggest that BIA-derived SMI should complement rather than replace more comprehensive body composition assessment when feasible, and that muscle quality metrics (radiodensity, intramuscular adipose tissue) and inflammatory biomarkers warrant incorporation for enhanced prognostic stratification. Prospective validation of serial BIA-derived SMI changes during systemic therapy may further clarify its utility for longitudinal prognostic assessment and identify optimal monitoring intervals in advanced NSCLC.

These findings have several potential clinical implications. First, these findings support further prospective evaluation of routine skeletal muscle mass assessment using BIA in male patients with advanced NSCLC as part of comprehensive prognostic evaluation. If validated in multi-center cohorts, AWGS-recommended SMI cutoffs may provide a risk-stratification tool for male patients with advanced NSCLC in settings where standardized BIA capacity exists. Identification of low SMI at diagnosis may help clinicians stratify patients for more intensive supportive care interventions, including nutritional support, exercise programs, and closer monitoring. Second, the sex-stratified nature of the SMI-survival association underscores the importance of sex-stratified analysis in sarcopenia research and clinical practice. Pooling males and females or adjusting for sex as a covariate may obscure potential sex-related heterogeneity and warrants further investigation. Future clinical trials targeting low skeletal muscle mass or related body-composition abnormalities may consider evaluating potential sex-specific treatment effects. Third, the validation of AWGS BIA cutoffs in our cancer population provides preliminary evidence supporting their potential clinical utility in patients with lung cancers, at least for males. The AWGS threshold of 7.0 kg/m^2^ may represent a reasonable starting point for risk stratification in male patients with advanced NSCLC; however, external validation in independent cohorts is required before clinical adoption. For females, the prognostic relevance of the AWGS female cutoff requires further evaluation in larger cohorts. Fourth, our findings highlight the need for mechanistic research to elucidate the biological basis of sex-stratified findings in the muscle-cancer relationship. Understanding these mechanisms may reveal novel therapeutic targets and inform the development of potentially sex-tailored interventions to prevent or reverse cancer-related muscle loss.

### Strengths of the study

This study has several strengths. First, we employed rigorous sex-stratified multivariable analysis with pre-specified adjustment sets based on EPV considerations to avoid overfitting. For males (191 patients, 106 events), the fully adjusted model (Model B) included age, stage, ECOG performance status, Charlson comorbidity index, and treatment type (EPV = 15.14). For females (79 patients, 39 events), the fully adjusted model included age, stage, and ECOG performance status (EPV = 9.75). Different adjustment sets were pre-specified based on EPV considerations to maintain adequate EPV (>9) and avoid overfitting. Second, we performed comprehensive sensitivity analyses including continuous SMI modeling, formal interaction testing, and exclusion of early deaths, all of which supported the robustness of our primary findings. Third, we conducted exploratory cutpoint analysis with bootstrap resampling to validate the AWGS cutoffs in our population, demonstrating near-perfect concordance for males. Fourth, we used standardized BIA protocols and established AWGS criteria, enhancing reproducibility and clinical applicability. Fifth, our cohort included consecutive patients with advanced NSCLC receiving systemic therapy, representing a clinically relevant population.

### Limitations

However, several limitations warrant explicit acknowledgment. First, the retrospective single-center design limits causal inference and generalizability to other healthcare systems and populations. Although we adjusted for an extensive set of pre-specified clinical confounders and performed sensitivity analyses including smoking status and serum albumin as a nutritional/inflammatory proxy, residual confounding by unmeasured variables such as dietary intake, objective physical activity, inflammatory cytokines (e.g., IL-6, CRP), and detailed cachexia staging cannot be excluded. Prospective studies incorporating these domains are warranted. Second, the relatively small female sample size (*n* = 79, 39 events) may have limited statistical power to detect associations in this subgroup. The wide confidence interval for the female hazard ratio indicates substantial uncertainty, and a true association cannot be definitively ruled out. Third, we assessed muscle mass only at baseline; longitudinal changes in SMI during treatment were not evaluated. Dynamic changes in muscle mass may provide additional prognostic information beyond baseline measurements. Fourth, we did not assess muscle function (e.g., grip strength, gait speed) or physical performance, which are integral components of sarcopenia diagnosis according to AWGS criteria. The AWGS diagnostic framework emphasizes combined assessment of muscle mass, strength, and physical performance; absence of grip strength or gait speed data precluded classification according to full consensus criteria and prevented investigation of how muscle functional measures might modify SMI-outcome relationships. We additionally note that the updated AWGS 2025 framework no longer treats physical performance measures such as gait speed as mandatory diagnostic components, although muscle strength assessment remains essential for formal sarcopenia diagnosis. Low muscle mass alone may not fully capture the functional consequences of sarcopenia. Fifth, treatment decisions were not randomized and may have been influenced by baseline characteristics, including muscle mass, introducing potential selection bias. Sixth, BIA measurements reflect a specific device (InBody 570) and standardized protocol; transferability to different BIA platforms or varied measurement conditions cannot be assumed. Finally, our cohort was limited to Chinese patients; findings may not generalize to other ethnic groups with different body composition profiles and muscle mass distributions. Most critically, this analysis lacks external validation. Replication in an independent advanced NSCLC cohort with comparable BIA methodology would substantially strengthen confidence in the proposed male cutpoint and establish generalizability.

## Conclusion

In conclusion, the present study identified an association between low skeletal muscle index measured by bioelectrical impedance analysis and poorer overall survival in male patients with stage III–IV NSCLC. The empirically derived male SMI cutpoint (7.0 kg/m^2^) closely aligned with the AWGS guideline, demonstrating concordance with the AWGS threshold in lung cancer populations. In women, no statistically significant association was detected; however, the female subgroup was limited in size and statistical power, and these sex-stratified findings should therefore be interpreted cautiously. If validated in prospective multi-center studies, integrating routine BIA into clinical practice may help identify patients at increased risk and provide a basis for individualized supportive care strategies. At present, however, clinical implementation should remain conservative pending such validation. These results, derived from a single-center retrospective cohort, are hypothesis-generating and require external validation in larger, multi-center cohorts before sex-stratified interpretation can be applied in clinical practice. Further research is needed to clarify the clinical significance of the observed sex-stratified findings and to evaluate whether potential sex-related heterogeneity exists in the relationship between muscle mass and cancer outcomes.

## Data Availability

The raw data supporting the conclusions of this article will be made available by the authors, without undue reservation.
