# Supplementary material for: Sex-stratified prognostic value of low skeletal muscle index in advanced non-small cell lung Cancer: a retrospective cohort study
Source: Front Nutr. 2026 Jul 20;13:1816649. doi: 10.3389/fnut.2026.1816649 (PMC13429470; doi:10.3389/fnut.2026.1816649)
Supplement: Supplementary file 1 [file Supplementary_file_1.pptx]

## Slide 1
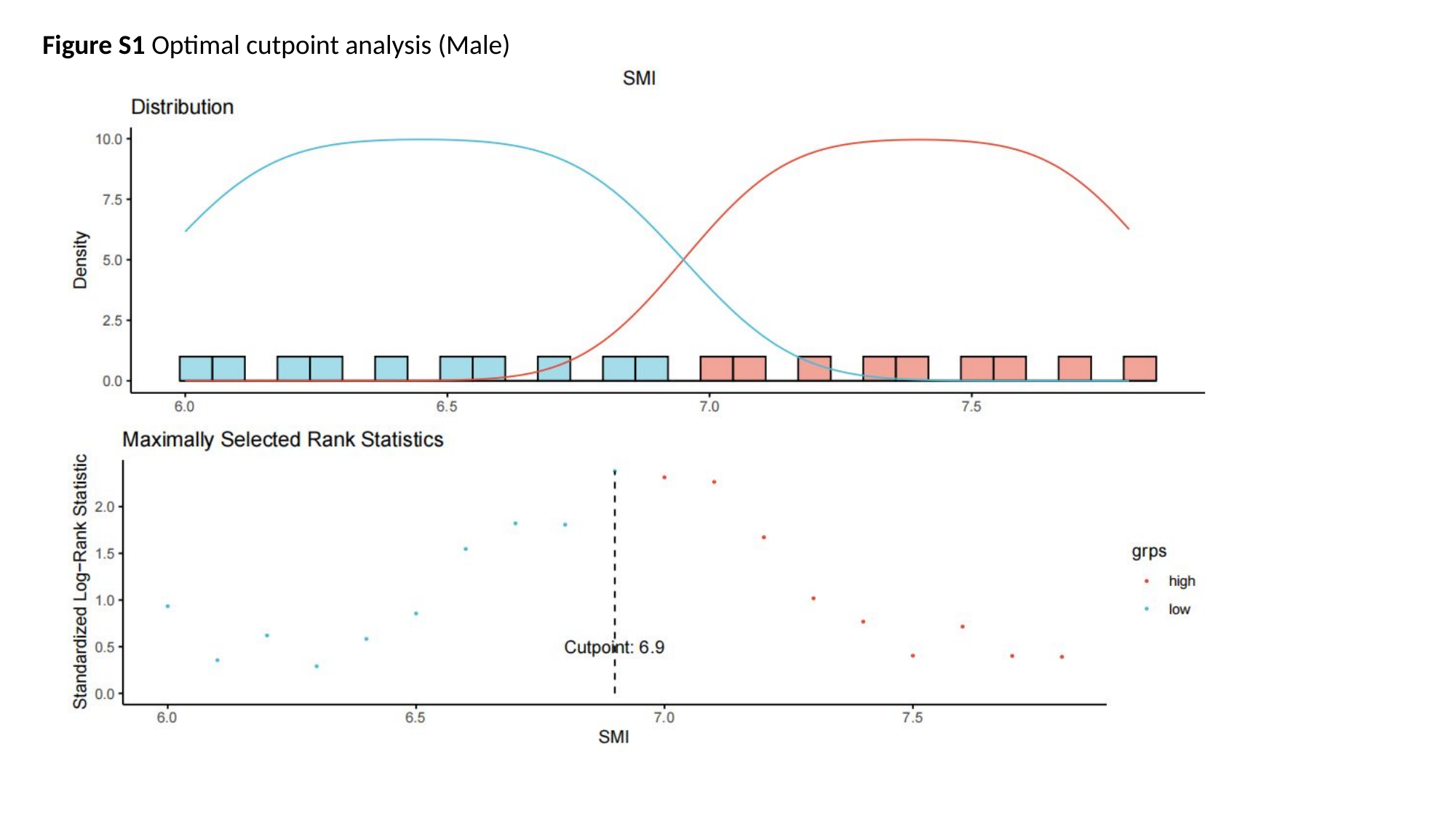

Figure S1 Optimal cutpoint analysis (Male)

## Slide 2
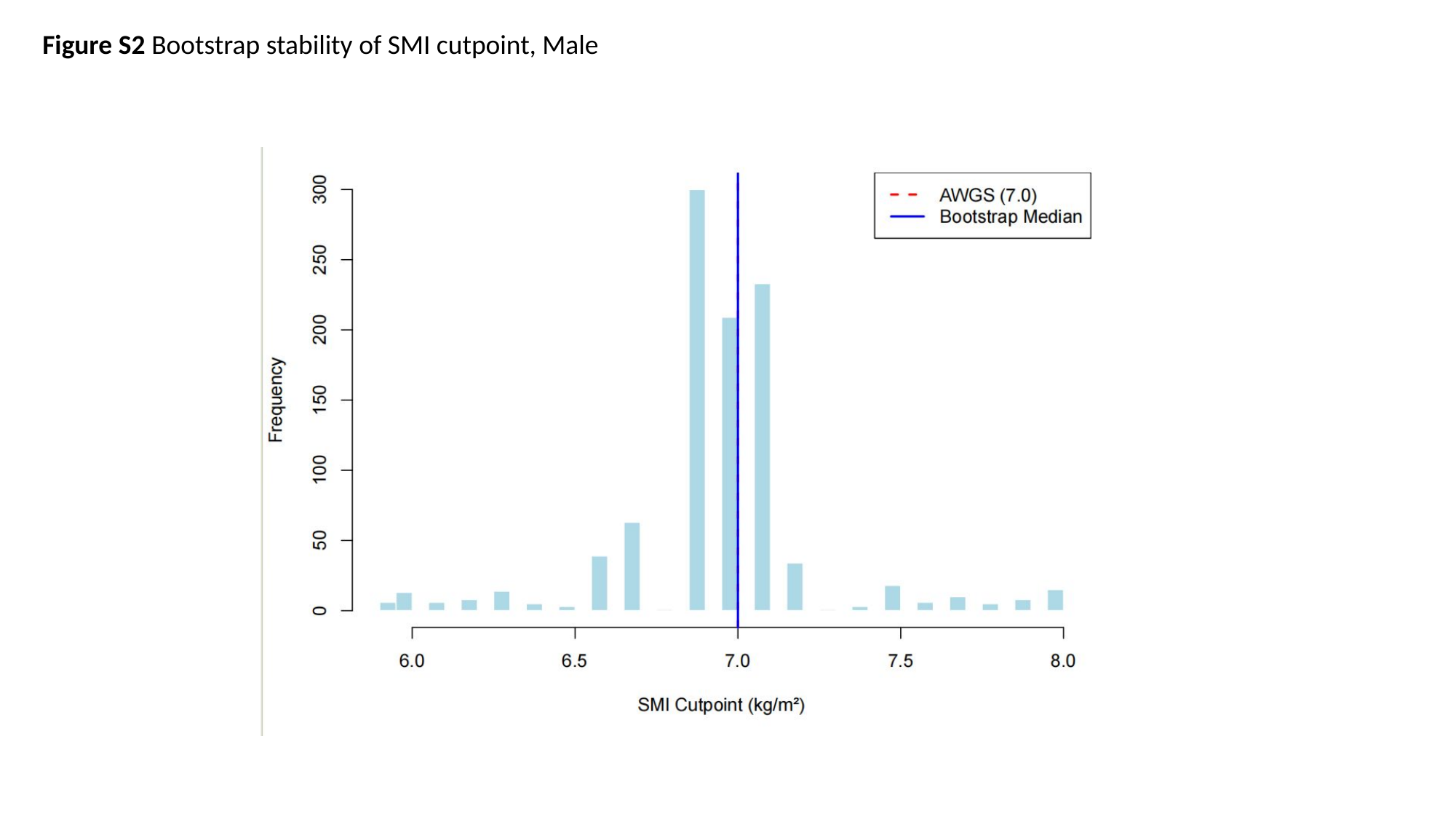

Figure S2 Bootstrap stability of SMI cutpoint, Male

## Slide 3
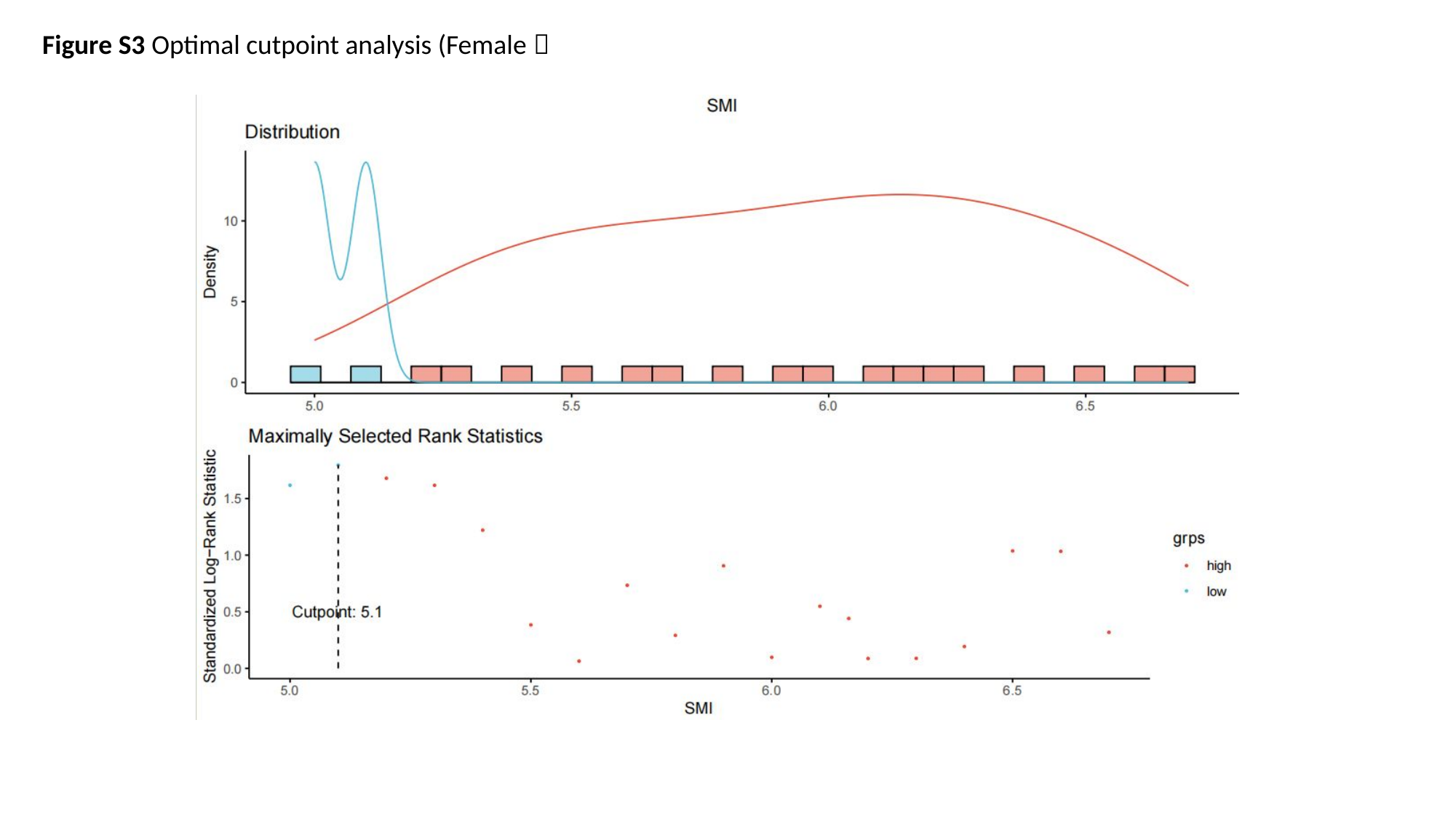

Figure S3 Optimal cutpoint analysis (Female）

## Slide 4
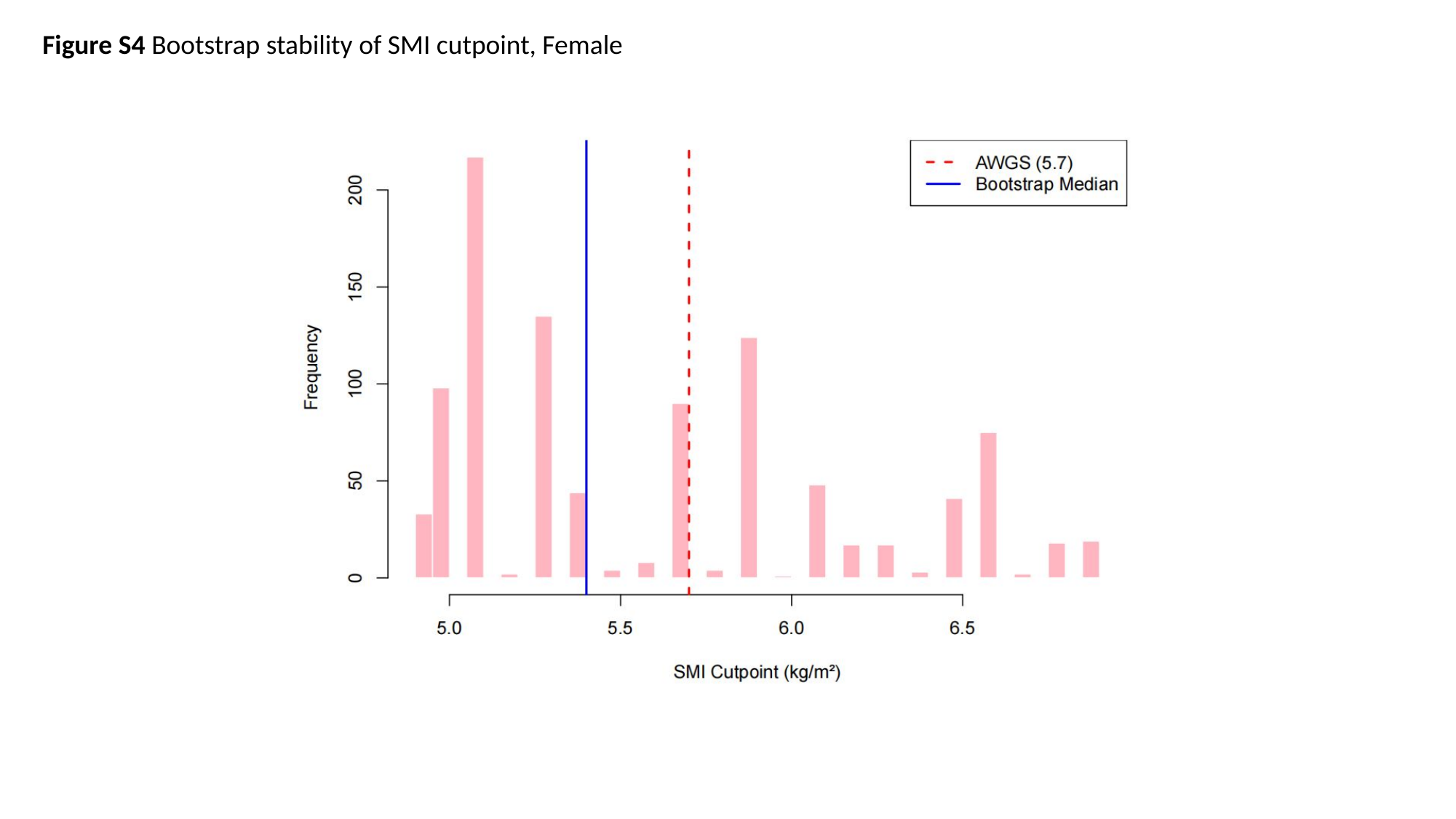

Figure S4 Bootstrap stability of SMI cutpoint, Female

## Slide 5
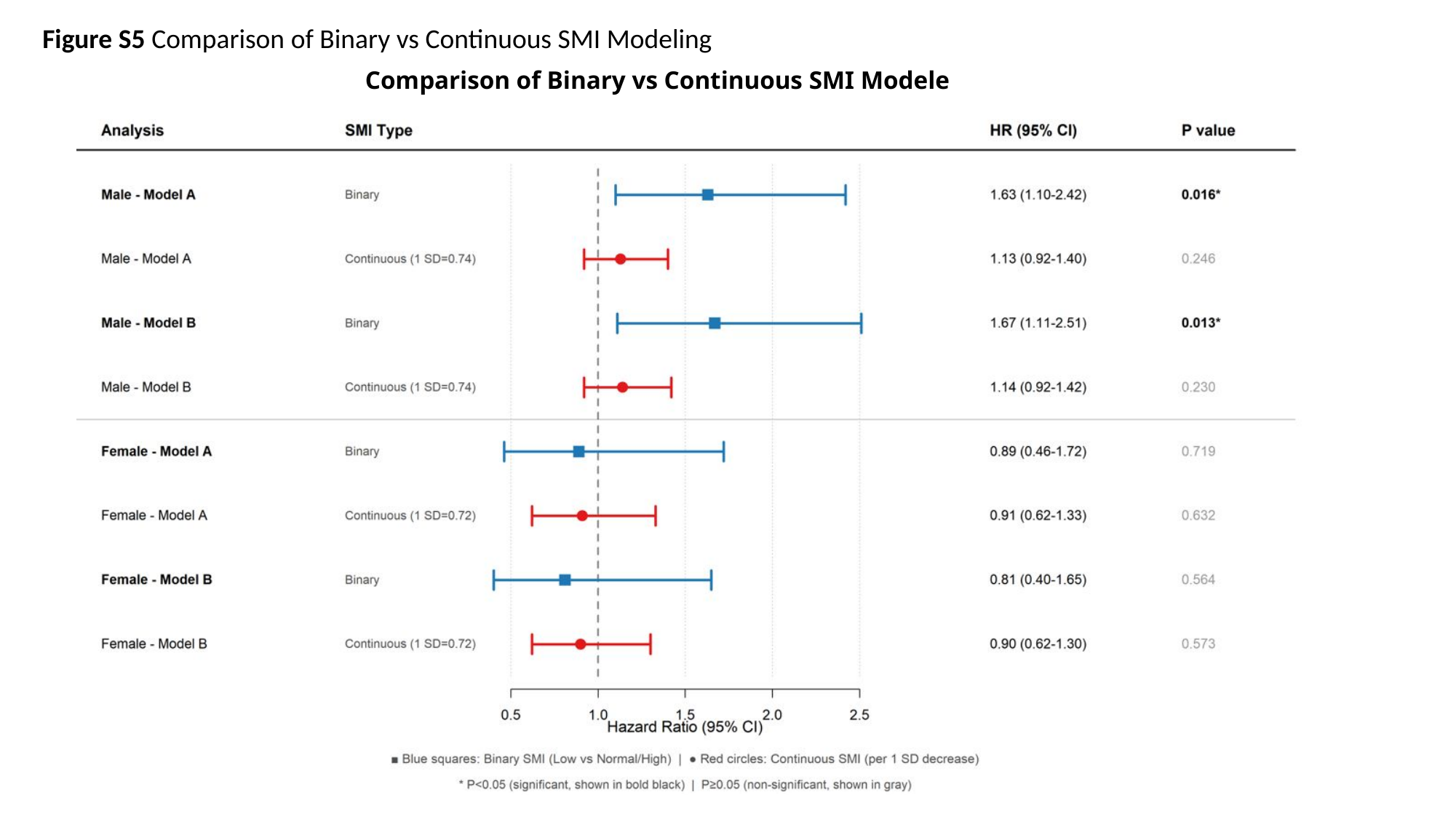

Figure S5 Comparison of Binary vs Continuous SMI Modeling
Comparison of Binary vs Continuous SMI Modele
