# Supplementary material for: Sex-stratified prognostic value of low skeletal muscle index in advanced non-small cell lung Cancer: a retrospective cohort study
Source: Front Nutr. 2026 Jul 20;13:1816649. doi: 10.3389/fnut.2026.1816649 (PMC13429470; doi:10.3389/fnut.2026.1816649)
Supplement: Supplementary file 2 [file Supplementary_file_2.docx]

SUPPLEMENTS

**Table S1** Bootstrap assessment of male SMI cutpoint (n = 191)

| **Parameter** | **Value** |
| --- | --- |
| Number of bootstrap runs | 1000 |
| Successful estimates | 1000 (100%) |
| Failed estimates (NA) | 0 |
| Median cutpoint (kg/m²) | 7.0 |
| 95% CI of cutpoint | 6.2 – 7.8 |
| AWGS guideline cutpoint | 7.0 |

Bootstrap resampling (1,000 iterations) was performed to assess the stability of the empirically derived cutpoint from maximally selected rank statistics. “Successful estimates” are iterations in which surv_cutpoint returned a valid cutpoint; all 1,000 iterations returned valid estimates. The median and empirical 95% CI (2.5th–97.5th percentiles) were derived from the distribution of bootstrap estimates. The AWGS guideline cutpoint (7.0 kg/m²) is shown for reference. SMI, skeletal muscle index; AWGS, Asian Working Group for Sarcopenia; CI, confidence interval.

**Table S2** Bootstrap assessment of female SMI cutpoint (n = 79)

| **Parameter** | **Value** |
| --- | --- |
| Number of bootstrap runs | 1000 |
| Successful estimates | 1000 (100%) |
| Failed estimates (NA) | 0 |
| Median cutpoint (kg/m²) | 5.4 |
| 95% CI of cutpoint | 4.9 – 6.8 |
| AWGS guideline cutpoint | 5.7 |

Bootstrap resampling (1,000 iterations) was performed as in Table S1. The empirical median cutpoint (5.4 kg/m²) was lower than the AWGS guideline (5.7 kg/m²), but the AWGS cutpoint lies within the bootstrap 95% CI (4.9–6.8 kg/m²), indicating moderate concordance with some uncertainty attributable to the smaller female sample size. For consistency with established guidelines and to facilitate clinical implementation, AWGS thresholds were retained for all analyses. SMI, skeletal muscle index; AWGS, Asian Working Group for Sarcopenia; CI, confidence interval.

**Table S3** Multivariable Cox Regression with SMI as Continuous Variable (per 1 SD Decrease)

| **Males** | **Model A** | **Model B** | **Females** | **Model A** | **Model B** |
| --- | --- | --- | --- | --- | --- |
| **Variable** | **HR**  **(95% CI)** | **HR**  **(95% CI)** | **Variable** | **HR**  **(95% CI)** | **HR**  **(95% CI)** |
|  | **P value** | **P value** |  | **P value** | **P value** |
| :--- | :--- | :--- | :--- | :--- | :--- |
| **SMI (per 1 SD decrease)** | 1.13 (0.92-1.40) | 1.14 (0.92-1.42) | **SMI (per 1 SD decrease)** | 0.91 (0.62-1.33) | 0.90 (0.62-1.30) |
| 1 SD = 0.74 kg/m² | P = 0.246 | P = 0.230 | 1 SD = 0.72 kg/m² | P = 0.632 | P = 0.573 |
| **Age (per year)** | 1.00 (0.98-1.03) | 1.00 (0.97-1.03) | **Age (per year)** | 1.05 (1.01-1.09) | 1.05 (1.01-1.09) |
|  | P = 0.928 | P = 0.890 |  | P = 0.007 | P = 0.008 |
| **Stage** |  |  | **Stage** |  |  |
| III | 1.00 (Ref) | 1.00 (Ref) | III | 1.00 (Ref) | 1.00 (Ref) |
| IV | 3.16 (1.76-5.68) | 2.25 (1.11-4.58) | IV | 2.26 (0.86-5.92) | 2.15 (0.81-5.70) |
|  | P < 0.001 | P = 0.025 |  | P = 0.099 | P = 0.125 |
| **ECOG Performance Status** |  |  | **ECOG Performance Status** |  |  |
| 0-1 | — | 1.00 (Ref) | 0-1 | — | 1.00 (Ref) |
| ≥2 | — | 1.43 (0.94-2.19) | ≥2 | — | 1.32 (0.64-2.72) |
|  |  | P = 0.098 |  |  | P = 0.460 |
| **Charlson Comorbidity Index** (per point) | — | 1.09 (0.96-1.23) | **Charlson Comorbidity Index** (per point) | — | — |
|  |  | P = 0.174 |  |  |  |
| **Treatment Type** |  |  | **Treatment Type** |  |  |
| A (Targeted) | — | 1.00 (Ref) | A (Targeted) | — | — |
| B (Immuno/Combined) | — | 1.20 (0.77-1.88) | B (Immuno/Combined) | — | — |
|  |  | P = 0.426 |  |  |  |
| C (Chemo/Radio) | — | 1.05 (0.54-2.07) | C (Chemo/Radio) | — | — |
|  |  | P = 0.881 |  |  |  |
|  | **Model A** | **Model B** |  | **Model A** | **Model B** |
| **Sample Size** | N = 191 | N = 191 | **Sample Size** | N = 79 | N = 79 |
| **Events** | 106 | 106 | **Events** | 39 | 39 |
| **C-statistic** | 0.608 | 0.643 | **C-statistic** | 0.656 | 0.655 |

HR, hazard ratio; CI, confidence interval; SD, standard deviation; ECOG, Eastern Cooperative Oncology Group; Ref, reference category. Model Aadjusted for age and stage. Model B adjusted for age, stage, ECOG performancestatus, Charlson comorbidity index (males only), and treatment type (males only).SMI modeled as continuous variable with negative coding such that HR > 1 indicates worse outcome with lower SMI. — indicates variable not included in the model.

**Table S4** Sex × SMI Interaction Tests in Combined Cohort

| **Analysis** | **Interaction Term** | **Wald P** | **LR Test P** |
| --- | --- | --- | --- |
| Binary SMI | SMI_AWGS × Sex | 0.120 | 0.115 |
| Continuous SMI | SMI_scaled × Sex | 0.551 | 0.554 |

Interaction models adjusted for age, stage, and ECOG performance status. SMI_AWGS: Low vs Normal/High based on AWGS cutoffs. SMI_scaled: Standardized continuous SMI (per 1 SD). LR, likelihood ratio. While formal interaction tests did not reach statistical significance (P<0.05), the trend in binary SMI interaction (P=0.120) combined with clear stratified differences supports the sex-stratified analytical approach.

**Table S5** Sensitivity Analysis Excluding Early Deaths (≤30 Days)

| **Sex** | **Model** | **Original Analysis** | **Sensitivity Analysis** | **N** | **Events** |  |  |
| --- | --- | --- | --- | --- | --- | --- | --- |
|  |  | **HR (95% CI)** | **P value** | **HR (95% CI)** | **P value** |  |  |
| Male | Model B | 1.67 (1.11-2.51) | 0.014 | 1.82 (1.19-2.78) | 0.006 | 181 | 96 |
| Female | Model B | 0.81 (0.40-1.65) | 0.564 | 0.81 (0.40-1.65) | 0.564 | 79 | 39 |

Model B adjusted for age, stage, ECOG performance status, Charlson comorbidity index (males only), and treatment type (males only). Ten early deaths (all male) were excluded in sensitivity analysis. Results in males were strengthened after excluding early deaths, demonstrating robustness. Results in females were unchanged as no early deaths occurred in this subgroup.

**Table S6. Sensitivity analyses of the multivariable Cox models for the association between low skeletal muscle index and all-cause mortality, by sex.**

| **Sex** | **Model** | **Adjustments beyond base covariatesᵃ** | **N** | **Events** | **HR**  **(95% CI)ᵇ** | ***P* value** |
| --- | --- | --- | --- | --- | --- | --- |
| **Male** | Model B (reference) | None (base covariates only) | 191 | 106 | 1.67 (1.11–2.51) | 0.013 |
|  | Model C | + Smoking status | 191 | 106 | 1.68 (1.11–2.52) | 0.013 |
|  | Model D | + Serum albumin | 189 | 106 | 1.58 (1.05–2.39) | 0.029 |
|  | Model E | + Smoking and albumin | 189 | 106 | 1.59 (1.05–2.40) | 0.029 |
| **Female** | Model B (reference) | None (base covariates only) | 79 | 39 | 0.81 (0.40–1.65) | 0.564 |
|  | Model D | + Serum albumin | 78 | 39 | 0.88 (0.43–1.79) | 0.726 |

AWGS, Asian Working Group for Sarcopenia; CI, confidence interval; ECOG, Eastern Cooperative Oncology Group; HR, hazard ratio; SMI, skeletal muscle index.

Data are hazard ratios (HRs) for low skeletal muscle index (SMI) versus normal/high SMI, with 95% confidence intervals (CIs) and two-sided *P* values from sex-stratified multivariable Cox proportional hazards regression. Low SMI was defined by Asian Working Group for Sarcopenia (AWGS) cut-points: <7.0 kg/m² for males and <5.7 kg/m² for females, measured by bioelectrical impedance analysis.

^a^ **Base covariates** (used in all models) included age (continuous, years), clinical stage (III vs IV), Eastern Cooperative Oncology Group (ECOG) performance status (0–1 vs ≥2), Charlson comorbidity index (continuous), and first-line treatment modality (targeted therapy / immunotherapy or combination / chemotherapy or radiotherapy). Model B is the fully adjusted multivariable Cox model reported in the main manuscript (Table 4); Models C–E sequentially add smoking status (current or former vs never) and/or serum albumin (continuous, g/L) as pre-specified sensitivity analyses.

^b^ In males, the SMI–mortality association was robust across all four models (HR range 1.58–1.68, all *P* ≤ 0.029). In females, the null association persisted after albumin adjustment.

Female Model C (smoking-adjusted) was not estimable because only 5 of 79 women (6.3%) were current/ever smokers, which produced sparse-data instability with grossly inflated standard errors; smoking was therefore excluded from the female model.

Sample size reductions in Models D and E (191→189 in males; 79→78 in females) reflect listwise deletion of missing serum albumin values (n=3 overall, 1.1%).

**Table S7. Post-hoc power analysis for the female subgroup (n = 79, 39 events).**

| **Assumed HR** | **Power (%)** | **Interpretation** |
| --- | --- | --- |
| 1.30 | 11.7 | Insufficient to detect small effect |
| 1.50 | 22.1 | Low power to detect moderate effect |
| 1.67 | 32.5 | Low power to detect male-observed effect |
| 2.00 | 53.0 | Moderate power to detect large effect |
| 2.50 | 76.7 | Adequate power to detect large effect |
| 3.00 | 89.7 | High power to detect very large effect |

HR, hazard ratio; power calculated using the Schoenfeld (1983) approximation for Cox proportional hazards models.

Post-hoc power calculated using the Schoenfeld (1983) approximation for Cox models. Female subgroup: n = 79, events = 39, low SMI prevalence = 32.9%; SMI_AWGS: Low vs Normal/High based on AWGS cutoffs. Observed HR = 0.81 (95% CI 0.40–1.65). While no statistically significant association was detected, this likely reflects limited power rather than absence of a true effect; replication in larger female cohorts is needed.
